# ﻿Three new ant-eating spiders of the family Zodariidae Thorell, 1881 (Araneae, Zodariidae) from Xishuangbanna, China

**DOI:** 10.3897/zookeys.1175.107644

**Published:** 2023-08-18

**Authors:** Ying Lu, Shuqiang Li, Hao Yu, Zhiyuan Yao

**Affiliations:** 1 College of Life Science, Shenyang Normal University, Shenyang 110034, Liaoning, China Shenyang Normal University Shengyang China; 2 Institute of Zoology, Chinese Academy of Sciences, Beijing 100101, China Institute of Zoology, Chinese Academy of Sciences Beijing China; 3 College of Chemistry and Life Sciences, Integrated Mountain Research Institute, Guizhou Education University, Guiyang, Guizhou, China Guizhou Education University Guiyang China

**Keywords:** Biodiversity, morphology, new species, taxonomy, Tropical Botanical Garden

## Abstract

Three new zodariid spiders from the Xishuangbanna Tropical Botanical Garden in Yunnan, China are described. A new species of the genus *Euryeidon* Dankittipakul & Jocqué, 2004, *E.dian***sp. nov.** (♂♀), and two new species of the genus *Mallinella* Strand, 1906, *M.banna***sp. nov.** (♂) and *M.mengla***sp. nov.** (♂), are described. *Euryeidon* is reported for the first time in China.

## ﻿Introduction

With 1265 described species in 90 genera, Zodariidae Thorell, 1881 is one of the most species-rich spider families (WSC 2023). Zodariids are small to medium-sized wandering or burrowing spiders; they are terrestrial, and often associated with soil debris and leaf litter, or found under stones or decaying logs (Barrion and Litsinger 1992). In Southeast Asia, 206 species of Zodariidae belonging to 13 genera have been recorded. Amongst these, the genus *Mallinella* Strand, 1906 exhibits the highest species diversity, with 146 species recorded, mainly distributed in Thailand, Malaysia, Indonesia, Vietnam and the Philippines (WSC 2023). Zodariidae is poorly studied in China, with only 57 species in nine genera, i.e., *Asceua* Thorell, 1887, *Cydrela* Thorell, 1873, *Heliconilla* Dankittipakul, Jocqué & Singtripop, 2012, *Heradion* Dankittipakul & Jocqué, 2004, *Mallinella*, *Storenomorpha* Simon, 1884, *Tropizodium* Jocqué & Churchill, 2005, *Zodariellum* Andreeva & Tystshenko, 1968 and *Zodarion* Walckenaer, 1826, having been reported from the country (WSC 2023).

*Mallinella* is the most speciose genus of Zodariidae, with 221 described species, having a very large distribution ranging from Senegal across the African continent, thence to tropical Asia, finally reaching the northern tip of Australia (Jocqué 1993, 1995, WSC 2023). *Mallinella* is characterized by the presence of a single row of short spines in front of the spinnerets. Currently, only 25 *Mallinella* spiders have been reported in China (WSC 2023). *Euryeidon* Dankittipakul & Jocqué, 2004, a small genus in Zodariidae, includes six species, and so far, has only been reported from Thailand (WSC 2023).

The Xishuangbanna Tropical Botanical Garden (XTBG) is managed by the Chinese Academy of Sciences, and is considered one of the most significant tropical rainforest nature reserves in Xishuangbanna, in southwestern Yunnan, China. Prior to the current study, three species of zodariid spiders had been described from Xishuangbanna: *Asceuamenglun* Song & Kim, 1997, *A.similis* Song & Kim, 1997 and *Mallinellalabialis* Song & Kim, 1997. In this paper, one new species of *Euryeidon* and two new species of *Mallinella* discovered within the XTBG are described. It is worth noting that the genus *Euryeidon* is recorded in China for the first time.

## ﻿Material and methods

Specimens were examined and measured with a Leica M205C stereomicroscope. Left male pedipalps were photographed. Epigynes were photographed before dissection. Vulvae were treated in a 10% warm solution of potassium hydroxide (KOH) to dissolve the soft tissues before illustration. Images were captured with a Canon EOS 750D wide zoom digital camera (24.2 megapixels) mounted on the stereomicroscope mentioned above and assembled using Helicon Focus v. 3.10.3 image stacking software (Khmelik et al. 2005). All measurements are given in millimetres (mm). Leg measurements are shown as: total length (femur, patella, tibia, metatarsus, tarsus), while missing data were coded as ‘*’. Leg segments were measured on their dorsal side. Spination pattern is listed for the prolateral, dorsal, retrolateral and ventral sides. The specimens studied are preserved in 75% ethanol and deposited in the Institute of Zoology, Chinese Academy of Sciences (**IZCAS**) in Beijing, China.

Terminology and taxonomic descriptions refer to Dankittipakul et al. (2010, 2012).

The following abbreviations are used in the descriptions:

**ALE** anterior lateral eye;

**AME** anterior median eye;

**AP** apical process of tegular apophysis;

**BPT** baso-prolateral tooth of tegular apophysis;

**d** dorsal;

**MOQ** median ocular quadrangle;

**MP** mesal process of tegular apophysis;

**MRF** meso-retrolateral fold of tegular apophysis;

**MRP** meso-retrolateral process of tegular apophysis;

**p** prolateral;

**PLE** posterior lateral eye;

**PME** posterior median eye;

**r** retrolateral;

**ST** subapical tooth of tegular apophysis;

**v** ventral.

## ﻿Taxonomy

### ﻿Family Zodariidae Thorell, 1881

#### ﻿Genus Euryeidon

Taxon classificationAnimaliaAraneaeZodariidae

Dankittipakul & Jocqué, 2004

694309C3-03F7-5748-B000-68D458100DE5

##### Type species.

*Euryeidonmonticola* Dankittipakul & Jocqué, 2004 from Thailand.

#### ﻿ Euryeidon dian

Taxon classificationAnimaliaAraneaeZodariidae

Lu & Li sp. nov.

A1E6F861-99C9-5F3B-9AE3-6A2B80F88702

https://zoobank.org//6E14D645-5552-4059-8253-17188C4F4ABC

Figs 1 , 2

##### Type material.

***Holotype***: 1♂ (IZCAS-Ar 44604), **China**, Yunnan, Xishuangbanna, Mengla County, Menglun Town, XTBG, Secondary tropical seasonal rain forest, 21°55.428′N, 101°16.441′E, 598 m, 16–31 June 2007, G. Zheng leg. ***Paratypes***: 1♂ (IZCAS-Ar 44605), 2♀ (IZCAS-Ar 44606, Ar 44607), same data as holotype, but secondary tropical seasonal moist forest, 21°54.54′N, 101°17.202′E, 713 m, 1–15 July 2007.

##### Etymology.

The specific name refers to the type locality (Dian is a short name for Yunnan) and is a noun in apposition.

##### Diagnosis.

**Figure 1.**
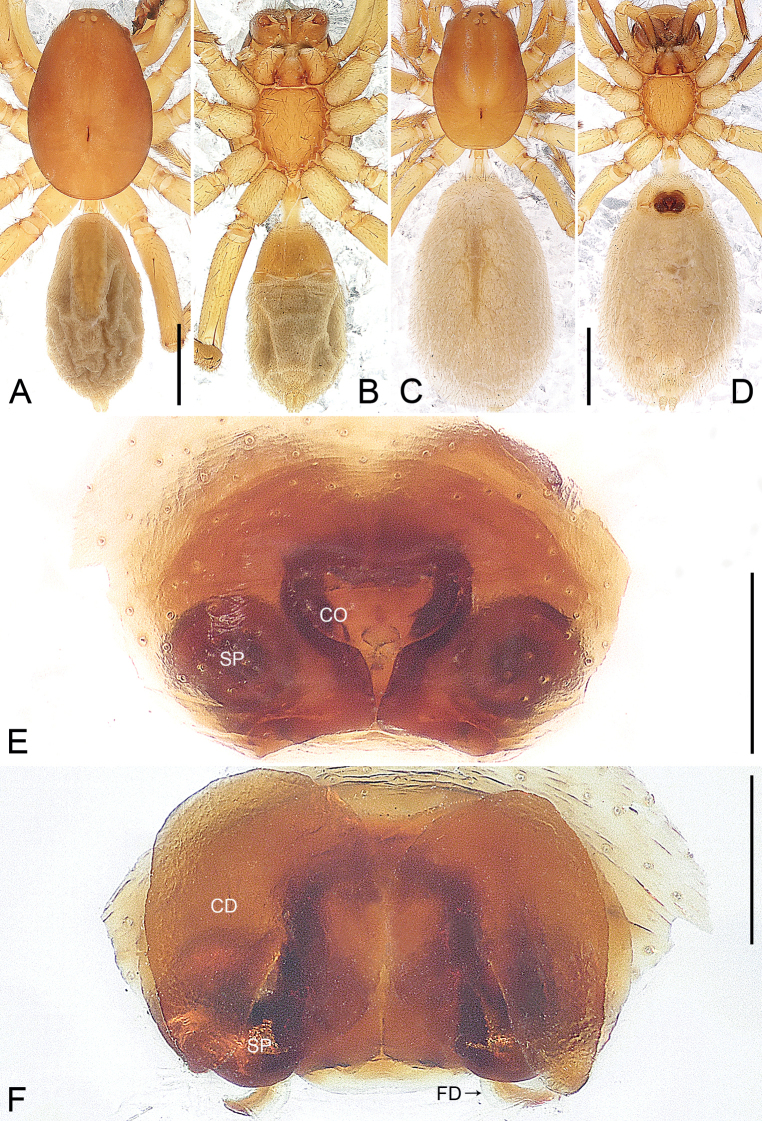
*Euryeidondian* sp. nov., holotype male (**A, B**) and paratype female (**C–F**) **A** habitus, dorsal view **B** habitus, ventral view **C** habitus, dorsal view **D** habitus, ventral view **E** epigyne, ventral view **F** vulva, dorsal view. Abbreviations: **CD** copulatory duct, **CO** copulatory opening, **FD** fertilization duct, **SP** spermatheca. Scale bars: 1.00 mm (**A–D**); 0.20 mm (**E, F**).

**Figure 2.**
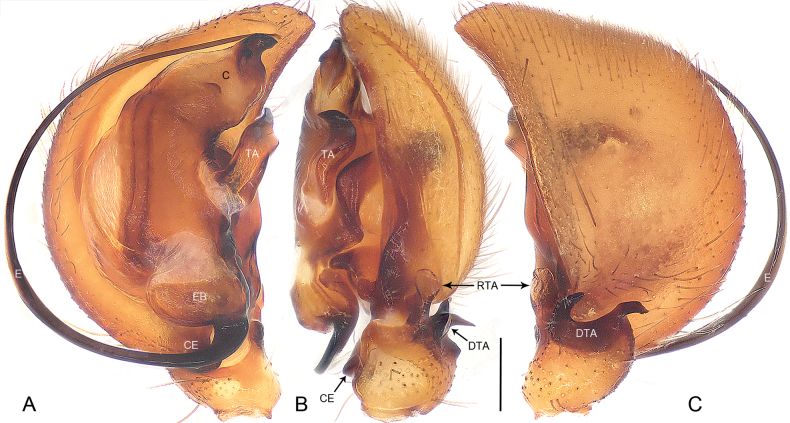
*Euryeidondian* sp. nov., holotype male **A–C** palp **A** ventral view **B** retrolateral view **C** dorsal view. Abbreviations: **C** conductor, **CE** cymbial extension, **DTA** dorsal tibial apophysis, **E** embolus, **EB** embolic base, **RTA** retrolateral tibial apophysis, **TA** tegular apophysis. Scale bar: 0.20 mm.

*Euryeidondian* sp. nov. resembles *E.sonthichaiae* Dankittipakul & Jocqué, 2004 (cf. Figs 1, 2 and Dankittipakul and Jocqué 2004: 763, figs 35–37) in that the males have a similar tegular apophysis (Fig. 2A, B). Males can be distinguished by the anterior part of the retrolateral tibial apophysis semi-transparent, and nearly round in ventral view (Fig. 2B, C; vs. retrolateral tibial apophysis strongly sclerotized, bifurcated, with sharply pointed apex), by the dorsal tibial apophysis bifurcated, with two curved and pointed apophyses (Fig. 2B, C; vs. dorsal tibial apophysis absent), by the longer filiform embolus (Fig. 2A, C; vs. embolus shorter), by the conductor being hook-shaped apically (Fig. 2A; vs. prolateral side of conductor greatly excavated to accommodate the embolic tip). Females can be easily distinguished from all known congeners by the bud-shaped copulatory openings (Fig. 1E), by the single-lobed spermathecae lying beneath the copulatory ducts (Fig. 1E), and by the copulatory ducts large, wing-shaped, and occupying the entire vulva (Fig. 1F).

##### Description.

**Male** (**holotype**; Fig. 1A, B). Total length 4.33; prosoma 2.05 long, 1.34 wide; opisthosoma 2.28 long, 1.07 wide. Eye sizes and interdistances: AME 0.04, ALE 0.08, PME 0.08, PLE 0.08, AME-AME 0.03, AME-ALE 0.12, PME-PME 0.05, PME-PLE 0.15; MOQ: 0.19 long, 0.11 anterior width, 0.19 posterior width. Leg measurements: I * (1.28, 0.55, 1.10, 0.89, *), II 4.02 (1.16, 0.55, 0.87, 0.72, 0.72), III 3.87 (1.06, 0.53, 0.72, 0.90, 0.66), IV * (1.28, 0.55, 1.06, 1.32, *). Spination: femora IV d100; tibiae I v122, II r11, v112, III p11, d010, r11, v222, IV p201, d110, r211, v222; metatarsi I p010, v222, II r010, v222, III p122, r122, v222, IV p112, d010, r112, v2222.

***Pattern and colouration*** (Fig. 1A, B). Carapace elongate oval, in profile flat, light brown, with distinct dark fovea, covered with pits. Chelicerae brown. Labium triangular, yellowish, apically with narrow membranous area and anteromedian scopula, basal and lateral margins slightly darker. Endites light brown, nearly triangular. Sternum light brown, shield-shaped, precoxal triangles and intercoxal sclerites present, anterior margin straight, with two small indentations at level of labium corner, posterior margin protruding. Legs yellowish, but brown on tarsus. Opisthosoma elongate oval. Dorsum of opisthosoma yellowish, with narrow band covering half of abdominal length. Venter yellowish. Spinnerets yellowish.

***Palp*** (Fig. 2A–C). Tibia with weakly sclerotized retrolateral tibial apophysis, anterior part semi-transparent, nearly round in ventral view; dorsal tibial apophysis strongly sclerotized, bifurcated, with two apophyses curved and sharply pointed. Cymbium with sclerotized prolateral extension and baso-retrolateral flange; cymbial fold approximately total length of cymbium. Tegular apophysis strip-shaped in ventral view, hooked in retrolateral view, sclerotized, situated on membranous base. Conductor sclerotized apically, hook-shaped, with small sub-apical tooth, posterior part membranous. Embolic base oval, originating posteriorly at 180°, attached to tegulum with membranous area anteriorly. Embolus filiform, long, curved and tapered apically.

**Female** (**paratype**; Fig. 1C, D). Total length 5.44; prosoma 1.92 long, 1.26 wide; opisthosoma 3.52 long, 1.86 wide. Eye sizes and interdistances: AME 0.03, ALE 0.07, PME 0.07, PLE 0.08, AME-AME 0.02, AME-ALE 0.13, PME-PME 0.05, PME-PLE 0.16; MOQ: 0.19 long, 0.07 anterior width, 0.17 posterior width. Leg measurements: I 3.98 (1.15, 0.54, 0.93, 0.70, 0.66), II 3.59 (1.00, 0.51, 0.75, 0.69, 0.64), III 3.36 (0.92, 0.49, 0.59, 0.80, 0.56), IV 4.75 (1.21, 0.52, 1.01, 1.15, 0.86). Spination: femora I–III d100, IV d11; patellae III–IV 101; tibiae I v122, II v11, III p11, d010, r11, v222, IV p11, d010, r111, v222; metatarsi I–II v222, III p112, r022, v222, IV p112, r112, v222.

***Pattern and colouration*** (Fig. 1C, D). Colour and somatic morphology as in male, except as noted. Dorsum of opisthosoma pale, without dorsal scutum and with light brown band centrally. Venter pale. Spinnerets pale.

***Genitalia*** (Fig. 1E, F). Epigynal plate fan-shaped and sclerotized. Copulatory openings bud-shaped, situated in the middle of epigynal plate. Vulva with round spermathecae, single lobed beneath the copulatory ducts, widely separate from each other, and with pair of fertilization ducts posteriorly, originating underneath the spermathecae. Copulatory ducts large, wing-shaped, occupying entire vulva.

##### Natural history.

The species was found in leaf litter.

##### Variation.

Male: total body length 4.14. Female: total body length 4.86.

##### Distribution.

China (Yunnan, type locality).

#### ﻿Genus Mallinella

Taxon classificationAnimaliaAraneaeZodariidae

Strand, 1906

693E696B-6360-59D9-B7A2-8A0B8B3862D8

##### Type species.

*Mallinellamaculata* Strand, 1906 from Ethiopia.

### ﻿The *hilaris* species group

#### ﻿ Mallinella banna

Taxon classificationAnimaliaAraneaeZodariidae

Lu & Li sp. nov.

8F22C6D3-3EB7-54F8-9790-15AD73E7597E

https://zoobank.org/1A39CD60-A034-4818-8C23-502EB5E87C38

Figs 3 , 4 , 7A

##### Type material.

***Holotype***: 1♂ (IZCAS-Ar 44612), **China**, Yunnan, Xishuangbanna, Mengla County, Menglun Town, XTBG, Primary tropical seasonal rain forest, 21°55.035′N, 101°16.500′E, 558 m, 4–11 May 2007, G. Zheng leg. ***Paratype***: 1♂ (IZCAS-Ar 44613), same data as holotype, but 21°57.669′N, 101°11.893′E, 790 m, 1–15 May 2007.

##### Etymology.

The specific name refers to the type locality and is a noun in apposition.

##### Diagnosis.

**Figure 3.**
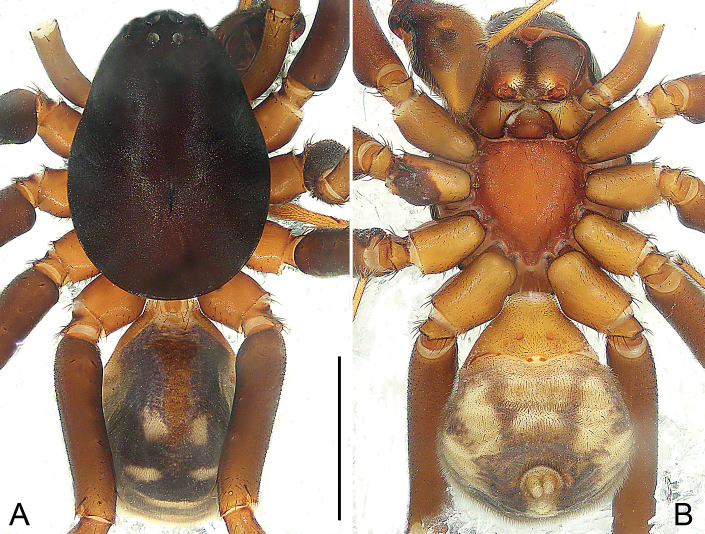
*Mallinellabanna* sp. nov., holotype male **A, B** habitus **A** dorsal view **B** ventral view. Scale bar: 2.00 mm.

**Figure 4.**
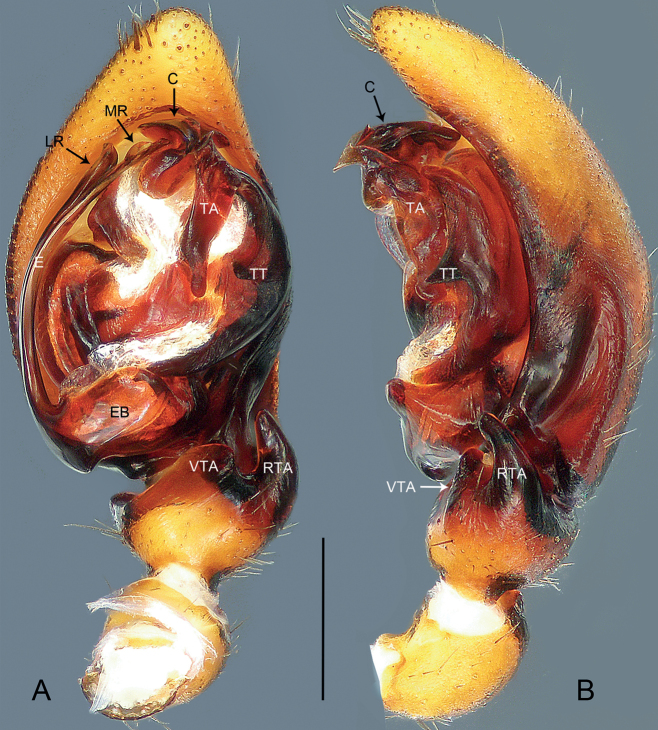
*Mallinellabanna* sp. nov., holotype male **A, B** palp **A** ventral view **B** retrolateral view. Abbreviations: **C** conductor, **E** embolus, **EB** embolic base, **LR** lateral ramus of embolus, **MR** mesal ramus of embolus, **RTA** retrolateral tibial apophysis, **TA** tegular apophysis, **TT** tegular tubercle, **VTA** ventral tibial apophysis. Scale bar: 0.50 mm.

*Mallinellabanna* sp. nov. resembles *M.sena* Lin & Li, 2023 (cf. Figs 3, 4, 7A and Lin et al. 2023: 97, figs 80A, B, 81A–C, 83A, B, 84I, J) in that the males have similar ventral tibial process, retrolateral tibial apophysis and tegular tubercle (Fig. 4A, B). Males can be distinguished by the mesal ramus of the embolus with a semicircular subterminal flange, apex lanceolate (Fig. 4A; vs. mesal ramus of embolus with longer, lanceolate apex), by the conductor pointing downwards, with lobular dorsal process, the prolateral side of conductor greatly excavated to accommodate embolic tip (Fig. 4A, B; vs. conductor pointing forward, dorsal process absent, excavation of conductor shallow prolaterally), and by the tegular apophysis undulate and indistinct, with thin, sharply pointed process apically, mesal ridge and baso-prolateral tooth absent (Figs 4A, B, 7A; vs. apical process of tegular apophysis wider, mesal ridge and baso-prolateral tooth present). Female unknown.

##### Description.

**Male** (**holotype**; Fig. 3A, B). Total length 6.48; prosoma 3.56 long, 2.52 wide; opisthosoma 2.92 long, 2.29 wide. Eye sizes and interdistances: AME 0.16, ALE 0.14, PME 0.13, PLE 0.14, AME-AME 0.11, AME-ALE 0.16, PME-PME 0.17, PME-PLE 0.58; MOQ: 0.49 long, 0.40 anterior width, 0.41 posterior width. Leg measurements: I 10.95 (2.64, 0.86, 2.50, 2.77, 2.18), II 9.47 (2.49, 0.93, 1.97, 2.42, 1.66), III 9.21 (2.36, 0.96, 1.74, 2.67, 1.48), IV 12.86 (3.17, 0.98, 2.71, 3.85, 2.15). Spination: femora I p001, d1111, II p011, d1111, III p011, d1111, r001, IV p101, d1111, r001; patellae II–IV 101; tibiae I p100, v222, II p11 v222, III p111, d11, r11, v222, IV p11, d11, r111, v2222; metatarsi I–II v222, III p11, d11, r111, v222, IV p21, r11, v22222.

***Pattern and colouration*** (Fig. 3A, B). Carapace pear-shaped, in profile highest between PME and longitudinal fovea; tegument rough and granulated, dark brown. Chelicerae brown. Labium triangular, yellowish to brown, apically with narrow membranous area, basal and lateral margins slightly darker. Endites brown, with anteromesal brush of black hairs. Sternum reddish-brown, shield-shaped, precoxal triangles and intercoxal sclerites present; anterior margin straight, protruding posteriorly. Legs yellowish, but brown on femur, with distinct dorsal swelling. Opisthosoma elongate oval, covered with numerous erect spines. Dorsum of opisthosoma dark brown, mottled with numerous pale spots, with narrow brown dorsal scutum anteriorly. Venter pale, with two pairs of dark stripes laterally. Posterior ventral spines thin and elongate, apices bluntly pointed, arranged in single row. Spinnerets yellowish.

***Palp*** (Fig. 4A, B). Tibia with fan-shaped ventral tibial process. Retrolateral tibial apophysis digitiform, broad at base, gradually tapering towards its bluntly pointed apex. Cymbial fold approximately 1/3 length of cymbium. Tegular tubercle triangular, sharply pointed. Tegular apophysis elongated, with thin, sharply pointed apical process (AP in Fig. 7A), also with small meso-retrolateral fold (MRF in Fig. 7A). Conductor beak-shaped, apex sharp, pointing downwards; prolateral side of conductor greatly excavated to accommodate embolic tip; dorsal process of conductor lobular. Embolic base oval, originating at 270°, with membranous area anteriorly. Embolus branching at half its length; mesal ramus thinner than lateral one, provided with semicircular subterminal flange, apex lanceolate; lateral ramus shorter.

##### Variation.

Male: total body length 5.95.

##### Natural history.

The species was found in leaf litter.

##### Distribution.

China (Yunnan, type locality).

### ﻿The *fronto* species group

#### ﻿ Mallinella mengla

Taxon classificationAnimaliaAraneaeZodariidae

Lu & Li sp. nov.

B56B5349-941E-5BF8-A889-AA0D68E3AD06

https://zoobank.org/5582D177-D171-4E8F-BF44-EFD96060F6C0

Figs 5 , 6 , 7B

##### Type material.

***Holotype***: 1♂ (IZCAS-Ar 44614), **China**, Yunnan, Xishuangbanna, Mengla County, Menglun Town, XTBG, 22°11.06′N, 100°39.05′E, 780 m, 23 May 2008, A. Weigel leg. ***Paratypes***: 2♂ (IZCAS-Ar 44615, Ar 44616), same data as holotype.

##### Etymology.

The specific name refers to the type locality and is a noun in apposition.

##### Diagnosis.

**Figure 5.**
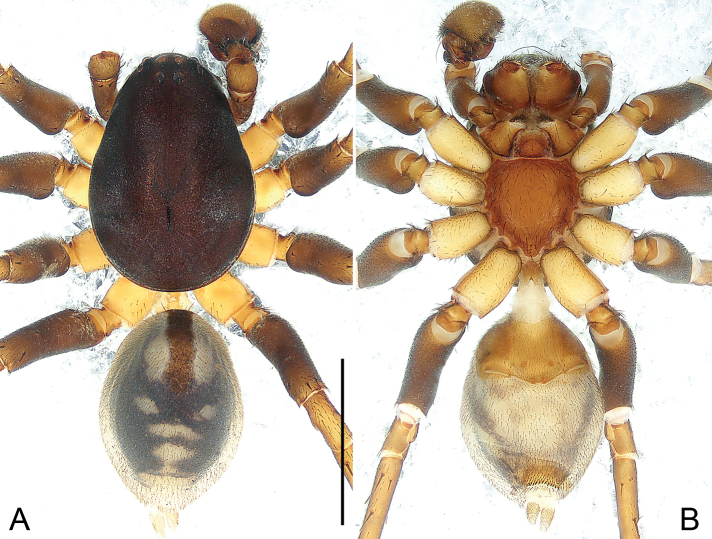
*Mallinellamengla* sp. nov., holotype male **A, B** habitus **A** dorsal view **B** ventral view. Scale bar: 2.00 mm

**Figure 6.**
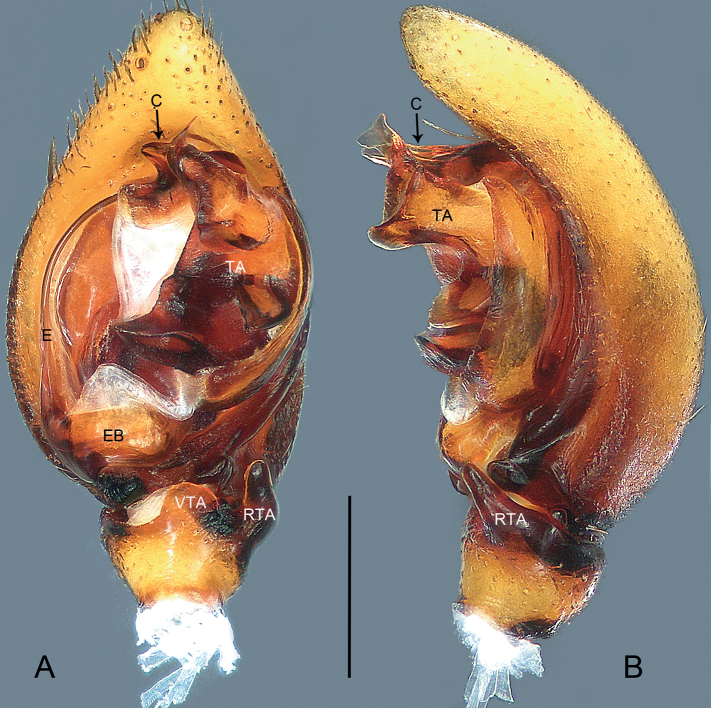
*Mallinellamengla* sp. nov., holotype male **A, B** palp **A** ventral view **B** retrolateral view. Abbreviations: **C** conductor, **E** embolus, **EB** embolic base, **RTA** retrolateral tibial apophysis, **TA** tegular apophysis, **VTA** ventral tibial apophysis. Scale bar: 0.50 mm.

**Figure 7.**
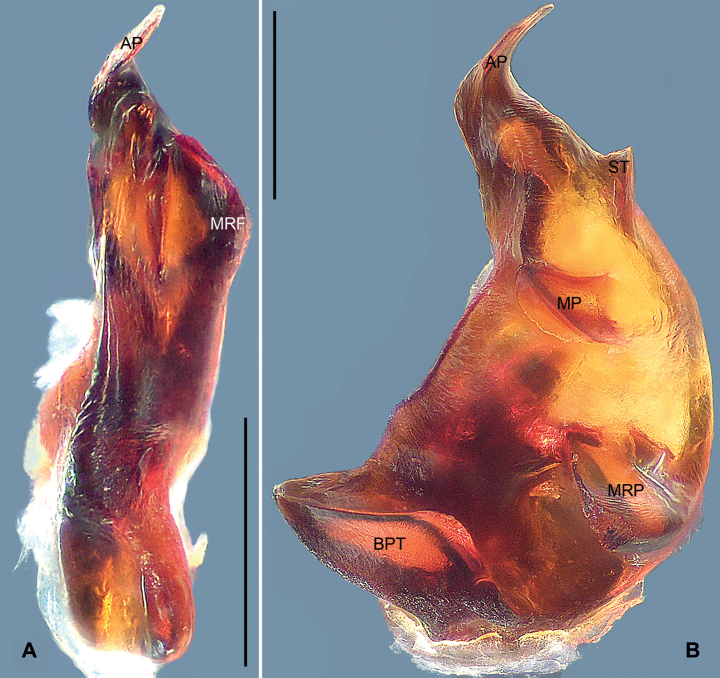
Ventral view of tegular apophysis, paratype male **A***Mallinellabanna* sp. nov. **B***Mallinellamengla* sp. nov. Abbreviations: **AP** apical process, **BPT** baso-prolateral tooth, **MP** mesal process, **MRF** meso-retrolateral fold, **MRP** meso-retrolateral process, **ST** subapical tooth. Scale bars: 0.20 mm.

*Mallinellamengla* sp. nov. resembles *M.stenotheca* Dankittipakul, Jocqué & Singtripop, 2012 (cf. Figs 5, 6, 7B and Dankittipakul et al. 2012: 262, figs 1113, 1187–1188, 1216–1223) in that the males have similar slender embolus and digitiform retrolateral tibial apophysis (Fig. 6A, B). Males can be distinguished by the conductor beak-shaped, with blunt apex, sclerotized extension prolaterally and lobular dorsal process (Fig. 6A; vs. conductor sitting in membranous area, with sharply pointed apex and dorsal sclerite), and by the tegular apophysis enormous, C-shaped in retrolateral view, with a thin, sharply pointed apical process in ventral view; subapical tooth triangular; medial ridge indistinct; mesal process elongated, digitiform in retrolateral view, apex blunt; meso-retrolateral process triangular in ventral view; baso-prolateral tooth thick and broad, directed anteriad, with blunt tip (Figs 6A, B, 7B; vs. tegular apophysis with elongated apical process, apex sharply pointed in lateral view; subterminal tooth triangular, situated anterior to blunt mesal tooth; baso-prolateral tooth broad, apex pointed in lateral view). Female unknown.

##### Description.

**Male** (**holotype**; Fig. 5A, B). Total length 5.83; prosoma 2.88 long, 2.00 wide; opisthosoma 2.95 long, 1.76 wide. Eye sizes and interdistances: AME 0.09, ALE 0.12, PME 0.11, PLE 0.13, AME-AME 0.10, AME-ALE 0.12, PME-PME 0.10, PME-PLE 0.22; MOQ: 0.37 long, 0.31 anterior width, 0.32 posterior width. Leg measurements: I 7.15 (1.86, 0.73, 1.63, 1.50, 1.43), II 6.57 (1.74, 0.75, 1.40, 1.43, 1.25), III 6.39 (1.64, 0.74, 1.21, 1.73, 1.07), IV 8.86 (2.16, 0.79, 1.84, 2.63, 1.44). Spination: femora I–III p001, d111, IV p001, d211, r001; patellae III–IV 101; tibiae I–II v222, III–IV p11, d11, r11, v222; metatarsi I–II v22, III p212, r212, v2222, IV p1111, r210, v2222.

***Pattern and colouration*** (Fig. 5A, B). Carapace pear-shaped, in profile highest between PME and longitudinal fovea; tegument rough and granulated, dark brown. Chelicerae brown. Labium triangular, yellowish to brown, apically with narrow membranous area, basal and lateral margins slightly darker. Endites brown, with anteromesal brush of black hairs. Sternum brown, shield-shaped, precoxal triangles and intercoxal sclerites present; anterior margin straight, protruding posteriorly. Legs yellowish, but brown on femur and femur I–III with distinct dorsal swelling. Opisthosoma elongated ovoid, covered with numerous erect spines. Dorsum of opisthosoma dark brown, mottled with numerous pale spots, with narrow brown dorsal scutum anteriorly. Venter pale, with one pair of dark stripes laterally. Posterior ventral spines thin and elongate, apices bluntly pointed, arranged in single row. Spinnerets yellowish.

***Palp*** (Fig. 6A, B). Tibia with fan-shaped ventral tibial process. Retrolateral tibial apophysis digitiform, relatively short, broad at base, gradually tapering towards its bluntly pointed apex. Cymbial fold approximately 1/4 length of cymbium. Tegular apophysis enormous, C-shaped in retrolateral view, with thin, sharply pointed apical process in ventral view (AP in Fig. 7B); subapical tooth triangular (ST in Fig. 7B); medial ridge indistinct; mesal process elongated, digitiform in retrolateral view, apex blunt (MP in Fig. 7B); meso-retrolateral process triangular in ventral view (MRP in Fig. 7B); baso-prolateral tooth thick and broad, directed anteriad, with blunt tip (BPT in Fig. 7B). Conductor beak-shaped, with blunt apex, sclerotized extension prolaterally and lobular dorsal process; base of conductor with sclerotized plate sitting in membranous area. Embolic base oval, originating at 270°, with triangular membranous area anteriorly. Embolus filiform and curved.

##### Variation.

Males: total body length 5.08–5.28.

##### Natural history.

The species was found in leaf litter.

##### Distribution.

China (Yunnan, type locality).

## ﻿Discussion

Adding the new species reported here, a total of six zodariid spider species are reported from XTBG. A checklist of XTBG zodariid spiders follows, and for a complete list of taxonomic references see WSC (2023).

1. *Asceuamenglun* Song & Kim, 1997 (♂♀)
2. *Asceuasimilis* Song & Kim, 1997 (♂♀)
3. *Euryeidondian* sp. nov. (♂♀)
4. *Mallinellabanna* sp. nov. (♂, the *hilaris* species group)
5. *Mallinellalabialis* Song & Kim, 1997 (♀, the *tuberculata* species group)
6. *Mallinellamengla* sp. nov. (♂, the *fronto* species group)

## Supplementary Material

XML Treatment for Euryeidon

XML Treatment for Euryeidon dian

XML Treatment for Mallinella

XML Treatment for Mallinella banna

XML Treatment for Mallinella mengla

## ﻿Additional information

### Author ORCIDs

Ying Lu https://orcid.org/0000-0002-0316-3564

Shuqiang Li https://orcid.org/0000-0002-3290-5416

Hao Yu https://orcid.org/0000-0002-9113-2425

Zhiyuan Yao https://orcid.org/0000-0002-1631-0949

### Data availability

All of the data that support the findings of this study are available in the main text.
